# Studying the role of axon fasciculation during development in a computational model of the Xenopus tadpole spinal cord

**DOI:** 10.1038/s41598-017-13804-3

**Published:** 2017-10-19

**Authors:** Oliver Davis, Robert Merrison-Hort, Stephen R. Soffe, Roman Borisyuk

**Affiliations:** 1 Brighton and Sussex Medical School, Brighton, UK; 2 School of Computing, Electronics and Mathematics, Plymouth University, Plymouth, UK; 3 School of Biological Sciences, University of Bristol, Bristol, UK; 4 Institute of Mathematical Problems of Biology – the Branch of Keldysh Institute of Applied Mathematics of Russian Academy of Sciences, Pushchino, 142290 Russia

## Abstract

During nervous system development growing axons can interact with each other, for example by adhering together in order to produce bundles (fasciculation). How does such axon-axon interaction affect the resulting axonal trajectories, and what are the possible benefits of this process in terms of network function? In this paper we study these questions by adapting an existing computational model of the development of neurons in the *Xenopus* tadpole spinal cord to include interactions between axons. We demonstrate that even relatively weak attraction causes bundles to appear, while if axons weakly repulse each other their trajectories diverge such that they fill the available space. We show how fasciculation can help to ensure axons grow in the correct location for proper network formation when normal growth barriers contain gaps, and use a functional spiking model to show that fasciculation allows the network to generate reliable swimming behaviour even when overall synapse counts are artificially lowered. Although we study fasciculation in one particular organism, our approach to modelling axon growth is general and can be widely applied to study other nervous systems.

## Introduction

In this paper we describe a computational model of the anatomy and functionality of neuronal networks in the spinal cord of the hatchling *Xenopus laevis* tadpole. This study is based on extensive previous research on axon growth^1–3^ and neuronal activity^4^ in this relatively simple and well-understood system. Our new growth model for finding the detailed neuronal connectivity (connectome) is based on a “developmental approach”: static dendrites are distributed along the spinal cord while growing axons, guided by chemical gradients, can produce synaptic contacts when they cross dendrites^3^. In this study we drastically revised the growth model such that all axons can grow simultaneously, which allows reciprocal interactions between them. This gives the possibility for growing axons to fasciculate (attract) or repulse each other. We assess the functionality of the network by mapping it onto a spiking functional model composed of single-compartmental Hodgkin-Huxley type neurons.

### Computational Modelling

Both the growth and functional models are biologically realistic and based on experimental data. The parameters of the growth model (without fasciculation) for each cell type are optimized such that they generate axonal patterns with the same statistical characteristics as experimentally measured axons. The parameters of the functional model have also been chosen according to the available electrophysiological data obtained from the different neuron types.

Our spinal cord model mimics the initiation of swimming in response to skin touch. It is known experimentally that trunk skin stimulation causes the neuronal circuits in the spinal cord to generate a pattern of spiking activity that corresponds to swimming, with left-right alternating motoneuron spikes at 10–25 Hz. The developmental process of axon growth and synapse formation generates connectomes which have specific properties that support swimming initiation. This circuit generates the swimming pattern in a very reliable way, even when a significant number of synapses are randomly deleted^4^.

### Fasciculation

Axonal fasciculation is the process of a growing axon adhering to another, potentially forming groups of axons known as bundles (“fascicles”), which follow similar growth trajectories. Gradient guidance is a common neurodevelopmental mechanism used to navigate growth cones during axonogenesis and this mechanism can include fasciculation. The fasciculation process depends on molecular interactions between proteins in axonal membranes (axolemmas), which can promote fasciculation, defasciculation (i.e. an axon leaving a bundle of fasciculated axons) or growth cone repulsion.

Fasciculation appears to be a common mechanism that has a wide range of different roles. For example, fasciculation helps glomerular formation by sensory neurons in the *Drosophila melanogaster* olfactory lobe, and guides retinotectal axon growth in vertebrates^5,6^. The importance of fasciculation is further illustrated by experiments in which the proteins that mediate it are mutated. For example, in *Xenopus laevis*, loss of the Neural Cell Adhesion Molecule (NCAM) that is expressed by sensory Rohon-Beard (RB) neurons causes a reduction in the RB population and its absence from the dorsolateral tract^7^. In *Drosophila melanogaster*, loss of fasciclin II, a vertebrate NCAM homologue, leads to loss of the vMP2 and MP1 fascicles, which are the first longitudinal tracts to develop in the *Drosophila* embryo^8^. Evidence from both *C*. *elegans* and human connectomes suggests that the formation of fascicles also helps to limit dispersion of axonal trajectories, reducing their average path length and producing more targeted connections between brain regions^9^.

Does loss of fasciculation have functional implications? The answer is unclear. The loss of fasciculation will most likely be a result of mutation of adhesive proteins, which likely have other biological roles; fasciclin II, for example, acts as a chemical signal^10^. This creates a difficulty in analysing the mechanistic contribution non-fasciculation makes towards a disease, because the disease may result from perturbation of the other functions of the mutated adhesive proteins. Nevertheless, some authors predict disease results from non-fasciculation. For example, the loss of vMP2 and MP1 fascicles in *Drosophila melanogaster* (due to mutated fasciclin II) predicted deleterious effects on correct synaptogenesis in the affected neuronal populations^8^. Furthermore, NCAM deficient mice exhibit mossy fibre fasciculation defects in the hippocampus, which is associated with reduced spatial learning and exploratory behaviour^11^. Aberrant synaptogenesis therefore represents a plausible mechanistic explanation for how loss of fasciculation could contribute to neurological disease. This is relevant to human diseases such as autism, which are associated with aberrant synaptogenesis^12^, and may help to explain why neurodevelopmental disorders arise in patients with NCAM mutations^13^. Given the potential importance of fasciculation to neurodevelopment, we attempt to computationally investigate the importance of its role in maintaining correct axon guidance.

Several computational models of axon growth attempt to represent the dynamic nature of the growth cone and its fasciculation behaviour. In^14^ fasciculation is modelled as a culmination of short and long range attractive cues, while in^15^ attractive short-range interactions between axons of different types (referring to axons which express different levels of adhesion proteins in their membranes) are considered. These models were used to investigate the likely mechanisms that influence fasciculation and the physical dynamics of the process. These models successfully reproduce the process of fasciculation and incorporate important biological features, such as different attraction strengths between different axon types or axon turnover, which matches a more realistic mechanism in development. However, neither studies the effects of fasciculation within a complex chemical growth environment involving long-range growth cues that can attract/repulse axons in multiple directions in order to generate complex axonal trajectories, nor are they based on and measured against biological data from real animals. A different approach was taken in a recent paper^16^, where the mechanism by which axons can fasciculate and defasciculate by a process termed “zippering” was studied; a biophysical model was used to determine certain properties of this process, such as the force of adhesion between axons.

When fasciculation is included in our growth model, the resulting axons have a structure that consists of multiple bundles. Varying the model parameters changes the number of bundles and their average size. This corresponds with experimental results from real animals, which show that commissural axons at earlier stages of spinal cord development (stage 28/29) are arranged into multiple bundles, which are located at certain positions along the spinal cord^17^. Our functional simulations show that when the number of synapses is reduced, networks with fasciculation are able to generate more reliable swimming activity than the networks without fasciculation.

The paper is organized in the following way: Section 2 describes the models, including our new parallel axon growth model and the spiking physiological model. Section 3 shows the results of our experiments with the models, including patterns of axonal projection and spiking activity. Finally, section 4 gives the conclusions of our modelling and compares our findings with the results of other models and available biological data.

## Model description

Our model of nervous system development is an improved version of the model that we have previously described^3,4^. We will briefly summarize the original model here, but for full details see the earlier papers.

**Figure 1:**
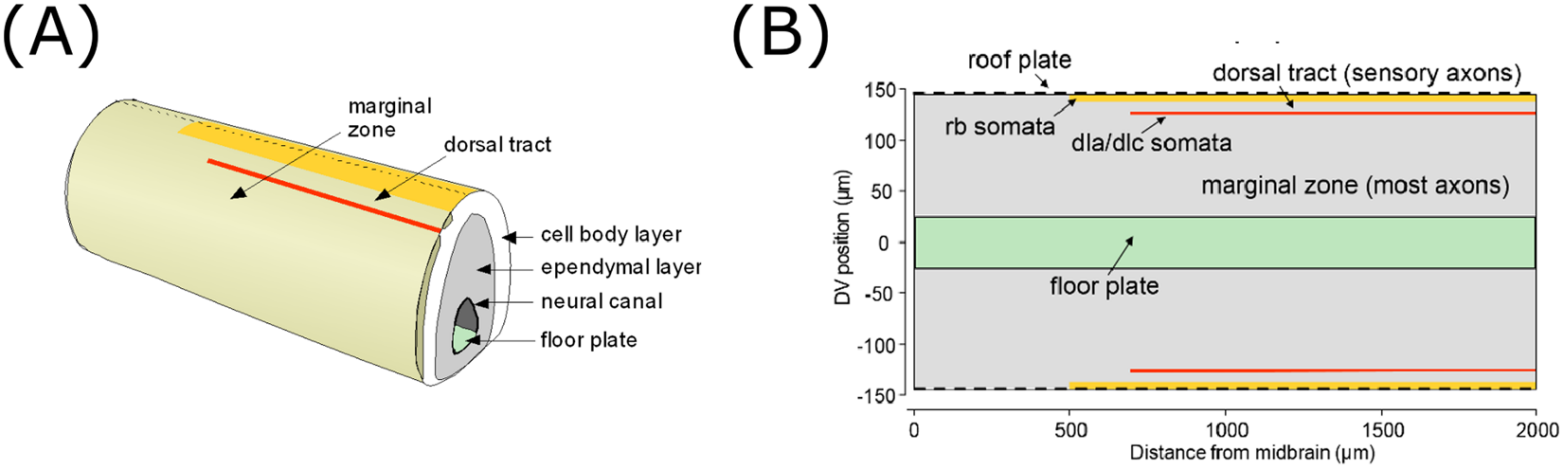
Overview of the tadpole spinal cord anatomy and the model’s 2D representation. (**A**) Diagram of a section of the tadpole CNS showing the main regions, including the neural canal surrounded by ependymal progenitor cells and the ventral floor plate, surrounded in turn by a layer of neuronal cell bodies. Lying outside the cell body layer is the marginal zone, in which most axons grow, and the dorsal tract which contains RB axons and is separated from the marginal zone by a column of sensory interneuron cell bodies (red line). Dorsally the marginal zone is bounded by a column of sensory RB neuron cell bodies (yellow line). (**B**) The CNS opened like a book along dorsal midline (dashed line in A) to show the two-dimensional modelling area.

We consider the spinal cord as a 2D rectangular area upon which somata, dendrites and axons are allocated (Fig. 1). This rectangle can be imagined as the result of opening the spinal cord like a book along the dorsal midline (dashed line in Fig. 1A). We disregard the third dimension (the thickness of the spinal cord “tube”) as this is very thin, with all somata, dendrites and axons located in a 10 µm thick region around the sides of the tube. The horizontal co-ordinate (*x*) corresponds to the distance from the tadpole’s midbrain in the rostro-caudal (RC) direction, and we only consider the region $500\,{\rm{\mu }}{\rm{m}} < {\rm{\times }} < {\rm{2000}}\,{\rm{\mu }}{\rm{m}}$. The vertical co-ordinate (*y*) corresponds to the distance from the ventral mid-line in the dorso-ventral (DV) axis, with positive values representing positions on the left side of the body and negative values the right. In this direction we only consider the region $-145\,{\rm{\mu }}{\rm{m}} < y < 145\,{\rm{\mu }}{\rm{m}}$. Although in this paper we only consider this short 1500 µm section of the spinal cord, when a longer section is considered the behavior is similar^4^.

There are seven different types of neuron included in the model: sensory Rohon-Beard (RB) neurons that respond directly to skin stimulation; dorso-lateral ascending and commissural neurons (dlas and dlcs) that make up the touch sensory pathway; ascending, descending and commissural interneurons (aINs, dINs, cINs) that make up the locomotor central pattern generator, and motoneurons (mns). In each generated connectome, the number of neurons of each type is fixed, with the same number of neurons of each type on both sides of the body. See Table 1 for neuron counts and the colour coding used throughout this paper.

**Table 1:**
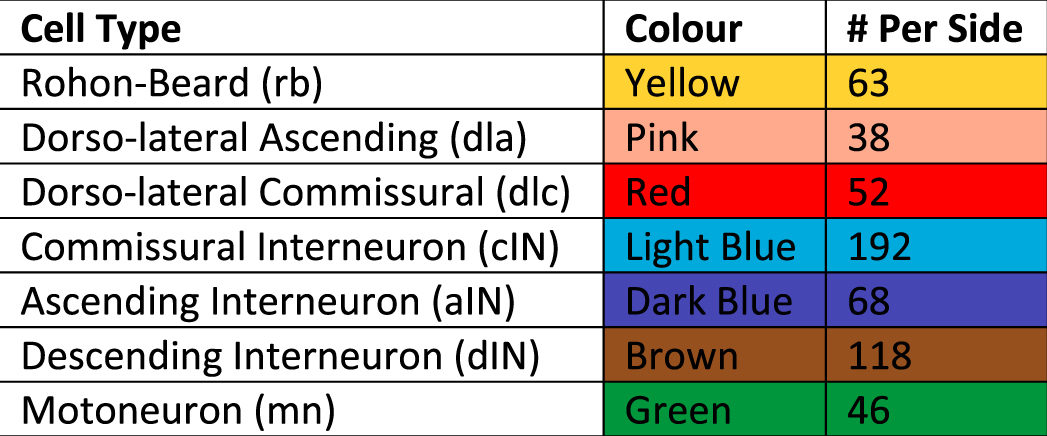
Cell types and their corresponding colours.

### Basic axonal growth procedure and model optimization

Axon growth in the model is based on two chemical gradients: one in the rostro-caudal direction and another in the dorso-ventral direction. The growth cone is characterized by different sensitivities to these gradients. Axons grow in discrete time with elongation ${\rm{\Delta }}=1\,{\rm{\mu }}m$ per time step. To describe the dynamics of the growing axon we use the growth angle ${\theta }^{A}$ which is characterised by “stiffness”, the tendency of the growing axon to grow straight, keeping the same growth angle which was used on the previous growth step. The influence of environmental cues (according to their gradients) deviates the growth cone from a straight path. The addition of a random variable at each step of growth provides an additional degree of freedom. Axon growth is guided by two gradients, resulting in a change of the growth angle value: One gradient, ${{G}}_{RC}$, influences the axon growth in the rostro-caudal direction, and the other, *G*_*DV*_, influences axon growth in the dorso-ventral direction. Each gradient is projected to the direction perpendicular to the current growth direction to describe the change of the growth angle. The model is described by the following difference equations:

1a $$x{}_{n+1}={x}_{n}+{\rm{\Delta }}\,\cos \,{\theta }_{n}$$

1b $$y{}_{n+1}={y}_{n}+{\rm{\Delta }}\,\sin \,{\theta }_{n}$$

1c $${\theta }_{n+1}^{A}={\theta }_{n}-{G}_{RC}({x}_{n},{y}_{n})\,\sin ({\theta }_{n})+{G}_{DV}({x}_{n},{y}_{n})\,\cos ({\theta }_{n})+{\xi }_{n}$$

1d $${\theta }_{n+1}^{B}=\{\begin{array}{c}{\theta }_{p},s > 0\\ {\bar{\theta }}_{p},s < 0\end{array}$$

1e $${\theta }_{n+1}=(1-|s|){\theta }_{n+1}^{A}+|s|{\theta }_{n+1}^{B}$$

Here $({x}_{n},{y}_{n},{\theta }_{n})$ are the current coordinates and growth angle of the axon tip at time step *n* and Δ is the axon elongation at each step (usually 1 µm). The term ${\theta }_{n}^{A}$ describes how the angle of growth changes according to rostro-caudal and dorso-ventral growth cues, as specified by the functions ${G}_{RC}(x,y)$ and ${G}_{DV}(x,y)$ respectively, as well as a random variable ${\xi }_{n}$ that is sampled at each step from a uniform distribution in the range $[-\alpha ,\alpha ]$. The parameter *s* and the term ${\theta }_{n+1}^{B}$ are used to model interactions between growing axons, and were not present in our previous model of axon growth. Parameter *s*, where $-1 < s < 1$, represents the sensitivity of axons to other nearby axons, while the term ${\theta }_{p}$ in equation 1d is an angle calculated from the growth angle of the closest nearby axon, as described below. Note that if $s=0$ then ${\theta }_{n+1}^{B}$ has no effect on the angle of growth and the model behaves identically to the previously published version.

To start axon growth, we assume that the initial axon position coincides with the soma position. The initial value of the growth angle is randomly selected using the 2D generalization procedure described in ^3^ for experimental measurements of pairs $(y,\theta )$, where *y* is the DV coordinate of the soma and *θ* is the initial angle of axon growth near the soma. The axon length is selected using the same procedure, using experimental measurements of total axon length.

The functions that control axon growth in response to gradients, ${G}_{RC}(x,y)$ and ${G}_{DV}(x,y)$, contain another set of parameters that determine the chemical gradient environment, and how axons grow in response to this. The values of the parameters which describe the gradient environment are independent of cell type and were selected according to a general biological knowledge on the distribution and properties of chemical gradients. For each cell type four parameters controlling growth are required: three giving sensitivities to gradients and one giving the range of the random deviation variable (*α*). To find values for these parameters we use a pattern-search optimization procedure to minimize a cost function, which takes into account how much the generated axons differ from experimentally measured axons in terms of their dorso-ventral distribution and tortuosity (“wiggliness”). For details of the cost function and optimization procedure see^3^. The optimization procedure was repeated in order to find optimal parameter values for each neuron type.

It is important to note that the experimental measurements of individual axons that were used for the optimization procedure were collected from different animals and therefore represent trajectories of single axons growing independently from each other. Consequently, these data cannot be expected to reflect any possible effects of fasciculation within individual animals. In the case of the axon growth model with fasciculation (or repulsion), multiple axons from a single animal should be used for fitting, however such experimental data from tadpole spinal cord axons is not currently available. Therefore, to generate axons in the case of fasciculation/repulsion we used the same parameter values that were found by the optimization procedure in the model without fasciculation. Re-running the fitting procedure to find the optimal chemical gradient parameters with fasciculation produced worse fits to the experimental data than without axonal interactions (see section “Model optimization with fasciculation”), but this is to be expected given the limitations of the biological data. However, multiple simulations (N = 12) confirmed that using parameter values optimized for fasciculation produced axon growth that was similar to that obtained with the original optimized values, without unrealistic axon growth features such as spontaneous changes of direction.

### Branching points and commissural neuron growth

The primary axon of most neuron types grows from the soma in an ascending direction (from tail to head); the exceptions being the dINs and mns which have descending (head to tail) primary axons. Neurons of most types also have secondary axons, which grow from a particular branching point in the opposite direction to the primary axon. The neuron types that do not have secondary axons are the dlas and mns, as well as some dINs. The coordinates of branching points are selected in a random way using the generalization procedure described above, using available experimental data on branching point locations for each neuron type.

The axons of commissural neurons (dlcs and cINs) start to grow on one side of the body and rapidly navigate in the ventral direction due to the strong influence of DV gradients. Upon reaching the floor plate they cross the body and change their sensitivity to the DV gradients. After crossing the ventral midline, instead of moving with the gradient they continue to grow on the contralateral side of the body against the DV gradient, with deviation to the ascending direction. Their secondary axons are positioned on the contralateral side and grow in the descending direction^17,18^.

Axon growth is limited by the rectangular modelling area, and also by two longitudinal barriers. The model contains two dorsal barriers: one formed by RB somata located at DV co-ordinate $y=\pm 137$ and spanning most of the rostro-caudal extent $x > 500$, and another formed by dlc/dla somata located at $y=\pm 127$ and spanning the range $x > 700$. Together these barriers form a dorsal tract which exclusively contains axons of RB neurons. Ventrally, another barrier that spans the entire length of the environment with DV co-ordinate $y=25$ prevents axons (except those of commissural neurons) from entering the floor plate and crossing to the other side of the body.

There is limited biological evidence on the process of branching and secondary axon growth after bifurcation. For example, it was found that in the *Xenopus* spinal cord commissural neurons axon branching occurs after crossing the floor plate and in response to external cues found there, including Slit-Robo signalling^19,20^. It is not known exactly when after bifurcation secondary commissural axonogenesis begins, although some data suggests it is soon after appearance of the branching point^21^. Mouse spinal cord sensory neurons possess secondary axons that similarly bifurcate in response to Slit-Robo signalling, and which grow soon after bifurcation^20^. In the model all primary axons grow first and then all secondary axons. We chose to do this because of the lack of concrete biological evidence of secondary axon growth timing, and will instead investigate the effects of varied secondary axon growth timings in future versions of the computational model.

### Dendrites and synapse formation

Each neuron has a dendritic field represented as a single line oriented in the dorso-ventral direction, centred on the soma position. The dendritic extents (i.e. length of the line) for each neuron are chosen using the same generalization process as axon initial angle and length, based on experimental measurements of dendritic fields of each neuron type. If a growing axon crosses a dendrite then synaptic connection will be generated with some probability. The probabilities of synaptic connections between neurons of different types have been experimentally defined in various pairwise recording experiments (for details see^1^). The “geography” of neurons has a strong impact on synaptogenesis. RB axons, for example, have *y*-coordinates in the range $127 < |y| < 137$ because they are allocated in the dorsal tract. Therefore, the axons of  RB neurons have no chance to cross the motor neuron dendrites, as these are located in very ventral positions.

### Fasciculation/repulsion of growing axons

Our new version of the growth model includes the possibility for growing axons to fasciculate (attract) or repulse each other. The parameter *s* controls sensitivity to other axons $(-1\,\le s\le 1)$. If $s=0$ then the model does not include fasciculation/repulsion and behaves as in the previous version. Conversely, if $s=\pm 1$ then gradients have no effect on axon growth. Positive values of $s$ correspond to fasciculation and negative values correspond to repulsion.

**Figure 2:**
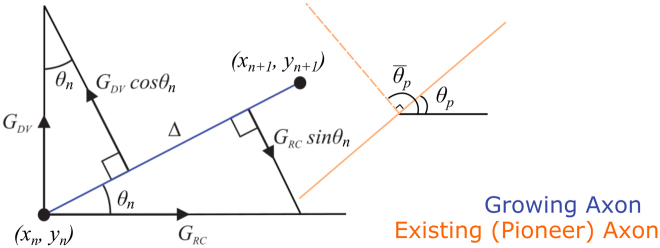
Schematic representation of the fasciculation/repulsion mechanism in the model. The solid orange line shows the direction of growth for the closest pioneer axon. This orange line is used to adjust the current growth angle of the follower axon shown by blue solid line. In case of fasciculation the current growth angle becomes closer to the growth angle of the pioneer axon. In case of repulsion the perpendicular dashed orange line is used to adjust the axon growth angle of the follower axon.

Figure 2 illustrates how the fasciculation/repulsion component of the growth angle is calculated. On the current step of growth the elongation from $({x}_{n},{y}_{n})$ to $({x}_{n+1},{y}_{n+1})$ is shown by the blue line and is determined by the growth angle ${\theta }_{n}$. To find the component of the growth angle corresponding to fasciculation or repulsion $({\theta }^{B})$ we consider the nearest existing axon (shown in orange) within a certain radius given by a parameter (*r*). The growth angle at the nearest point on the existing axon is denoted ${\theta }_{p}$, and the angle perpendicular to this is denoted ${\bar{\theta }}_{p}$. In case of fasciculation $(s > 0)$ the current axon will tend to grow with the same angle as the existing axon, whereas in the case of repulsion it will try to grow perpendicularly away from it.

While our previous model grew axons one at a time sequentially, the new version of the model allows axons from all neurons to grow at the same time, since this is required for axons to interact with each other. At each time step the growth cone positions of all axons are updated and each axon is elongated by the space step Δ. The growth process runs until all axons have been generated up to their full length. Different axons can begin growing at different times. For simplicity, we consider two groups of axons: “pioneers”, which grow first, and “followers”, which start to grow after all pioneers have finished.

### Assumptions/restrictions of anatomical model implementation

This paper presents a model based on anatomical measurements of developing *Xenopus* spinal cord neurons. We have attempted to incorporate as many biological details as possible into the model. However, there is no available anatomical information concerning fasciculation in animals at this stage of development, and there are still many open questions awaiting answers from experimental data. As a result, we have to formulate some assumptions for the model, some of which are motivated by logical considerations and some for the purposes of model simplification.

Assumption 1. Primary axons first, secondary after. We consider growth of primary axons and secondary axons as two consecutive processes. All primary axons grow first, and after that secondary axon growth starts. Naturally we prescribe a starting time for growth of each secondary axon. A future version of the model may include the possibility of starting secondary axon growth before the primary axon has finished growing.

Assumption 2. Fasciculation/synapses of commissural neurons on the opposite side only. We assume that the commissural neurons (dlcs and cINs) can fasciculate/repulse and create synaptic contacts on the contralateral side only. At the beginning, commissural neurons grow in the ventral direction according to the axon growth equations (1) with specially adjusted parameter values. After crossing the boundary of the ventral plate on the opposite side $(y=\pm 25)$ the axon growth is described by the same equations but with another regular set of parameter values^3^. During the initial pre-crossing stage the axons grow without fasciculation/repulsion and do not produce synapses. This assumption allows us to model axon growth on each side of the body independently of each other.

Assumption 3. Interaction with axons of the same cell type only. We assume that axons can fasciculate/repulse on to/away from the other axons (either primary or secondary) of the same type only (e.g. axons of cIN neurons can only fasciculate onto axons of other cINs). Although the biological reality is likely to be more complicated than this, we hypothesise that in many cases it is important for axons of a particular type to follow a specific patterns of growth, in order to achieve the macroscopic connectivity between populations required for proper network function. We would expect that if all neuron types were able to fasciculate onto each other, network connectivity would become less type-specific, which we would expect to negatively affect the network’s function. This hypothesis should be tested in future work.

Assumption 4. Universal values of fasciculation sensitivities. The fasciculation/repulsion sensitivity parameter has the same value for all cell types. However, we allow primary and secondary axons to have different sensitivities, denoted *s*_*pr*_ and *s*_*se*_ respectively.

Assumption 5. Fasciculation to the nearest point of a pioneer axon. A growing axon’s growth is only affected by a single point on an existing axon – specifically the point that is closest to its current growth cone position and within distance *r*. This point could be part of either a primary or secondary axon. At the next step of growth a new closest point is selected independently of the previous step.

### Functional model

The connectome produced by the growth model provides detailed information on connectivity in the spinal cord – specifically a list of synaptic connections between neurons. We use this information to build a functional model that simulates the spiking activity of spinal cord neurons. Briefly, this model is of single compartment Hodgkin-Huxley type, with channel properties chosen to match known electrophysiology. The functional model includes axonal and synaptic delays, as well as electrical coupling between dINs. For full details, see^4^. In contrast to the previous paper where custom software was used, in this paper the functional model simulations were performed using NEURON^22^ with a step size of 0.01ms. All other details of the model were identical to those previously published.

## Results

In this section we present the results of simulations of the growth and functional models. For the growth model we first show typical patterns of axon distribution for different neuron types in the case without fasciculation or repulsion. We then compare these results with the patterns that appear in the cases of fasciculation and repulsion. Similarly, we analyse how these different patterns of axonal projection affect synaptic connectivity. We then use the connectomes resulting from these growth simulations to produce spiking activity patterns using the functional model. Here, one of the main aims is to investigate possible functional roles of fasciculation.

### Connectome with fasciculation

#### Axon patterns for growth with fasciculation: general consideration

In this section we present the results of multiple simulations of the growth model with fasciculation or repulsion. Here, and in the following section, we focus on presenting qualitative results of our simulations. For each set of parameter values used, at least 12 connectomes were generated and the results compared to ensure they were visually similar. We look at more quantitative measures of the effects of fasciculation in the later sections. There are several parameters that control the effects of axonal interactions in the model (see Methods section). The sensitivity parameter *s* controls the strength of attraction/repulsion between a growing axon and an existing pioneer axon. When $s=0$ axons do not interact, whereas if $0 < s\le 1$ axons will fasciculate, and if  $-1\le s < 0$ they will repulse each other. The model includes two sensitivity parameters, *s*_*pr*_ and *s*_*se*_, for primary and second axons respectively. The range parameter *r* determines the maximum distance (in µm) from a growing axon tip over which an existing axon can exert an influence. Finally, for each neuron the time at which both its primary and secondary axons begin growing are specified. These parameters allow us to divide axons into two groups: pioneers that start to grow first, and followers which start to grow after the pioneers. Taken together, these parameters have a large effect on the resulting pattern of axon growth; for example, increasing the sensitivity parameter value results in axon bundles appearing.

**Figure 3:**
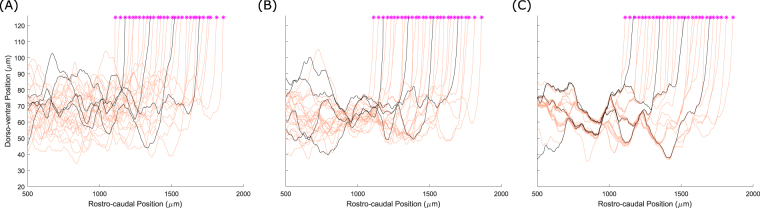
Patterns of axons for dla neurons. (**A**) Pattern without fasciculation. (**B**) Pattern with a relatively weak fasciculation $({s}_{pr}=0.1)$. (**C**) Pattern with ${s}_{pr}=0.5$, showing multiple axon bundles. The four pioneer axons are shown in black. The initial point of axon growth is shown for each neuron by a magenta star.

Figure 3 shows a set of dla axons on the left side of the tadpole body with various strengths of fasciculation: ${s}_{pr}=0$ (Fig. 3A), ${s}_{pr}=0.1$ (Fig. 3B) and ${s}_{pr}=0.5$ (Fig. 3C); in all cases $r=1\,{\rm{\mu }}{\rm{m}}$. In these simulations there are four pioneer axons (black lines in Fig. 3B,C) that start to grow simultaneously. All other axons (followers) grow after all the pioneer axons have finished growing. The time at which follower axons begin growing varies rostro-caudally, with the most rostral axon starting first and the most ventral axon last, with 200 time units between the start of each axon’s growth. One time unit corresponds to the real time which is needed for an axon to elongate by 1 µm (we assume that all axons grow uniformly with the same speed). Thus, when the length of the first follower axon is 200 µm the second follower starts to grow. Figure 3B clearly demonstrates that fasciculation, even with a small sensitivity value, leads to the axons grouping. The bundles are clearly visible in Fig. 3C where the value of sensitivity is even higher.

**Figure 4:**
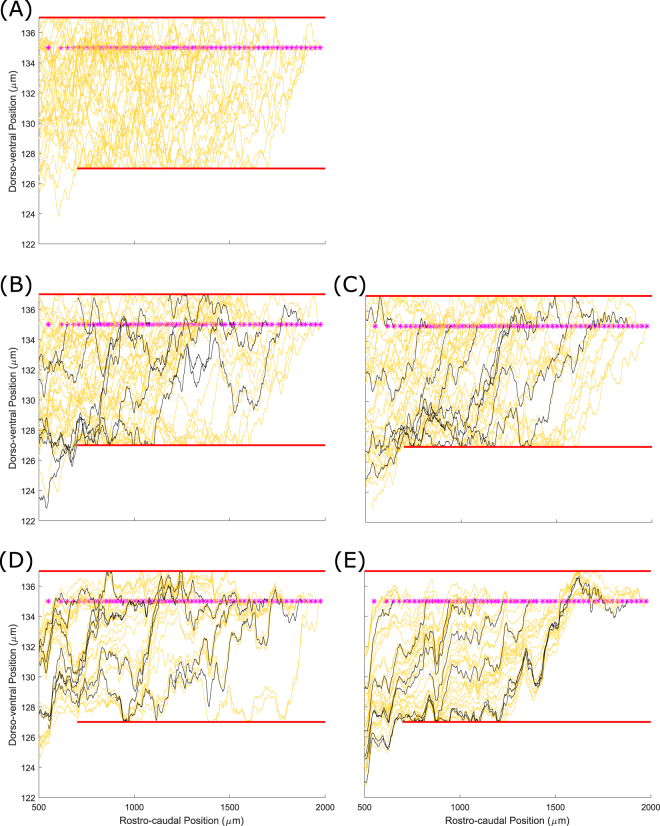
Patterns of primary sensory RB axons for different parameter values. (**A**) Primary RB axons without fasciculation; (**B**,**C**) Primary RB axons with sensitivity 0.1 and range 1 and 3 respectively; (**D**,**E**) Primary RB axons with sensitivity 0.5 and range 1 and 3 respectively. The initial point of axon growth is shown for each neuron by a magenta star.

Now we vary two parameters of the model: sensitivity and range. To illustrate how these parameters influence the pattern of axon growth we consider primary RB axons only. Figure 4(A) shows these axons without fasciculation. Red longitudinal bars show the barriers of the dorsal tract where all RB cells are positioned. The upper barrier is $(500 < x < 2000,\,y=137)$ and the lower barrier is $(700 < x < 2000,\,y=127)$. Figure 4(B),(D) shows the RB axon patterns for sensitivity ${s}_{pr}=0.1$ and ${s}_{pr}=0.5$ respectively. The range of attraction is 1 µm in both cases. Figure 4(C),(E) shows the patterns of RB axons for the same sensitivities ${s}_{pr}=0.1$ and ${s}_{pr}=0.5$ respectively. The range of attraction is 1 µm in both cases. Figure 4(C),(E) shows the patterns of RB axons for the same sensitivities and ${s}_{pr}=0.5$ respectively, but here the range is 3 µm. In all cases with fasciculation there are nine pioneer axons and their initial RC coordinates are equally spaced. These pioneer axons are shown by black lines.

Comparing Fig. 4(B),(D), with different sensitives and the same range, shows again that higher sensitivities give stronger groupings of axons. The same result is clear from comparing Fig. 4(C),(E). Although we expected that increasing the range at which axons can interact would increase the degree of fasciculation, this was not true for our model, as can be seen by comparing Fig. 4(B,D) ($r=1\,{\rm{\mu }}{\rm{m}}$) with (C, E) ($r=3\,{\rm{\mu }}{\rm{m}}$). The reason for this is clear: in our model, a positive value for the sensitivity means that neurons that are within range of each other will tend to grow in the same direction, not towards each other. This means that for larger values of the range (e.g. 3 μm) axons tend to grow in parallel with gaps between them, rather than bundling closely together. For the rest of this paper we avoid using such large values of the range, and instead fix $r=1\,{\rm{\mu }}{\rm{m}}$.

#### Patterns of primary and secondary axons

In this section we consider patterns of both primary and secondary axons for different values of the sensitivity parameters, with the range parameter fixed as 1 µm. Starting times for primary axons were defined in the same way as described in the previous section. When all primary axons have reached their full length, the secondary axons start to grow. The starting times of growth for secondary axons are chosen in a similar way to the primary axons. There are nine pioneer secondary axons which start to grow simultaneously, and after that all other secondary axons start to grow in sequence from rostral to caudal positions with a 200 time unit step. In the figures in this section, secondary axons are shown in magenta, to distinguish them from primary axons (yellow). Black lines show the pioneer axons.

**Figure 5:**
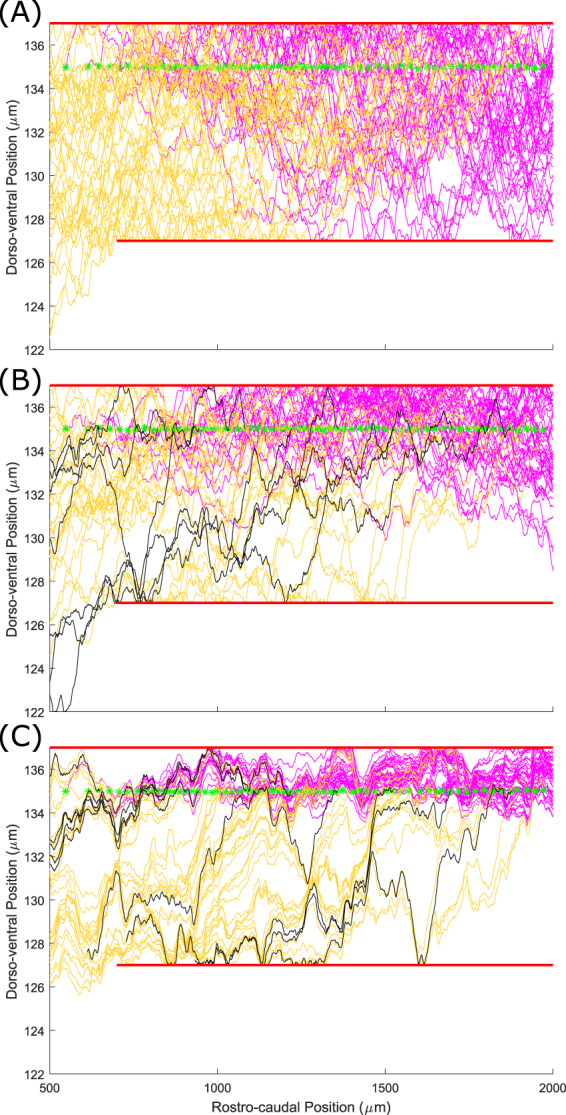
Patterns of sensory RB primary (yellow) and secondary (magenta) axons for different parameter values. Black lines show primary axons of pioneer neurons. (**A**) Primary and secondary RB axons without fasciculation; (**B**) Primary and secondary RB axons with sensitivity 0.1. (**C**) Primary and secondary RB axons with sensitivity 0.5. Note that while the primary axons (yellow) are equivalent to those shown in Fig. 4, the two figures are from different simulations, demonstrating the stochastic variability present in the model. Green stars show the initial point of the secondary axons.

Figure 5(A–C) shows three patterns of RB axons: (A) without fasciculation; (B) weak attraction ${s}_{pr}={s}_{se}=0.1$; and (C) strong attraction for ${s}_{pr}={s}_{se}=0.5$. It is clear in Fig. 5(A) that without fasciculation axons spread inside the dorsal tract and uniformly fill the space between two barriers: primary axons are located preferentially in the rostral part of the body and secondary ones in the caudal part. In the case of weak fasciculation (B) the pattern of axons is less dense and a tendency for axons to organise into groups is visible. Also, the secondary axons are more concentrated in the upper part of the simulated space near the higher barrier. Increasing the sensitivity value (C) makes this feature even clearer: several bundles of primary axons are visible and the secondary axons are located in the narrow area near the upper barrier.

**Figure 6:**
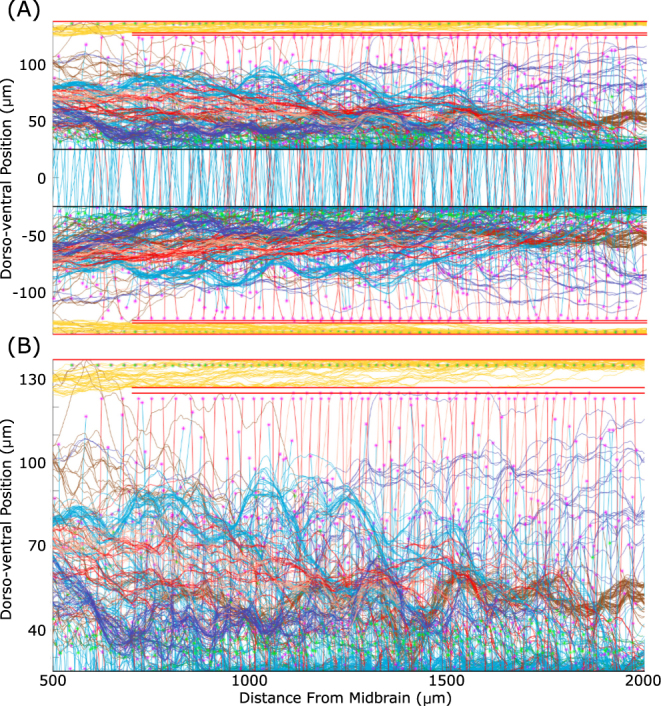
Full simulated spinal cord with all neuron types in the presence of fasciculation. (**A**) All axons in a fasciculated connectome with ${s}_{pr}={s}_{se}=0.2,r=1$, colour coded as in Table 1. (**B**) Zoom showing only axons on the left side of the body. Magenta and green stars show the initial point of the primary and secondary axon respectively. Note that the horizontal scale is the same in both figures.

In Fig. 6 we show all axons for the full connectome with fasciculation sensitivities ${s}_{pr}={s}_{se}=0.2$ and $r=1$. Figure 6(A) shows the axons on both sides and Fig. 6(B) is a zoom of the left side. These figures clearly show multiple axon bundles. Also, it is clear that the majority of axons (except those of RB neurons) are concentrated in the ventral part of the marginal zone. The density of axons near the floor plate is very high.

**Figure 7:**
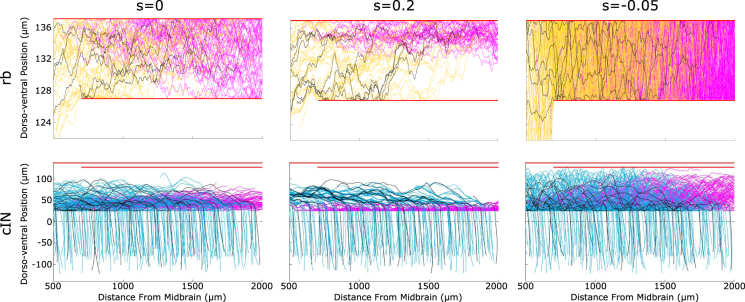
Patterns of axon growth for different neuron types with attraction, repulsion or neither. The left column shows axon patterns for RB (top) and cIN (bottom) cell types without fasciculation. The middle column shows axon patterns for the same cell types with fasciculation (sensitivity 0.2). The right column shows axon patterns for the same cell types in the case of repulsion (sensitivity −0.05). Primary axons are coloured according to Table 1, secondary axons are magenta and pioneer primary axons are black. Red horizontal lines indicate the boundaries of the marginal zone, and the dashed horizontal line in the bottom row corresponds to the ventral midline. cIN axons start on one side, cross the midline and then turn to grow longitudinally. Note that the horizontal scale is identical in all parts.

Simulations of the anatomical model show that variation of the sensitivity parameter value results in very different patterns of axon growth with fasciculation/repulsion. To illustrate this we present, for two example neuron types, RBs and commissural interneurons (cINs). For each type we show the pattern of primary and secondary axons without fasciculation, with fasciculation ${s}_{pr}={s}_{se}=0.2$, and with repulsion ${s}_{pr}={s}_{se}=-0.05$ (Fig. 7). It is clear from these figures that in case of fasciculation (middle column) the pattern of axons is much more concentrated than without fasciculation (left column), and multiple axon bundles are visible, particularly in the case of cINs. The cIN axons (especially secondary ones) are more concentrated in the ventral region when fasciculation is present, since axons that would otherwise grow to more dorsal positions are influenced to turn to grow longitudinally by those that have already grown, such as pioneers. In the case of repulsion (right column), even a small value of the sensitivity coefficient causes growing axons to grow in extremely tortuous trajectories that occupy all the available space. We would therefore expect repulsion to be associated with a decrease in the specificity of connections between types resulting in a loss of network function.

#### Fasciculation reduces the number of synapses

Our simulations show that the total number of synapses decreases when fasciculation is present. For example, when fasciculation sensitivity is 0.2 and range is 1 µm, the mean total number of connections is 72, 554 ± 753 (n = 12), compared with $\mathrm{81},\mathrm{877}\pm {\rm{744}}$ (n = 300) without fasciculation. Despite the overall reduction in synapse counts, the actual impact of fasciculation varies based on the type of the pre- and post-synaptic neuron, as Table 2 shows. Each entry gives the average (rounded) number of synapses between different cell types for connectomes with fasciculation $(s=0.2,r=1)$ and without fasciculation (first and second number in each cell respectively).

**Table 2:**
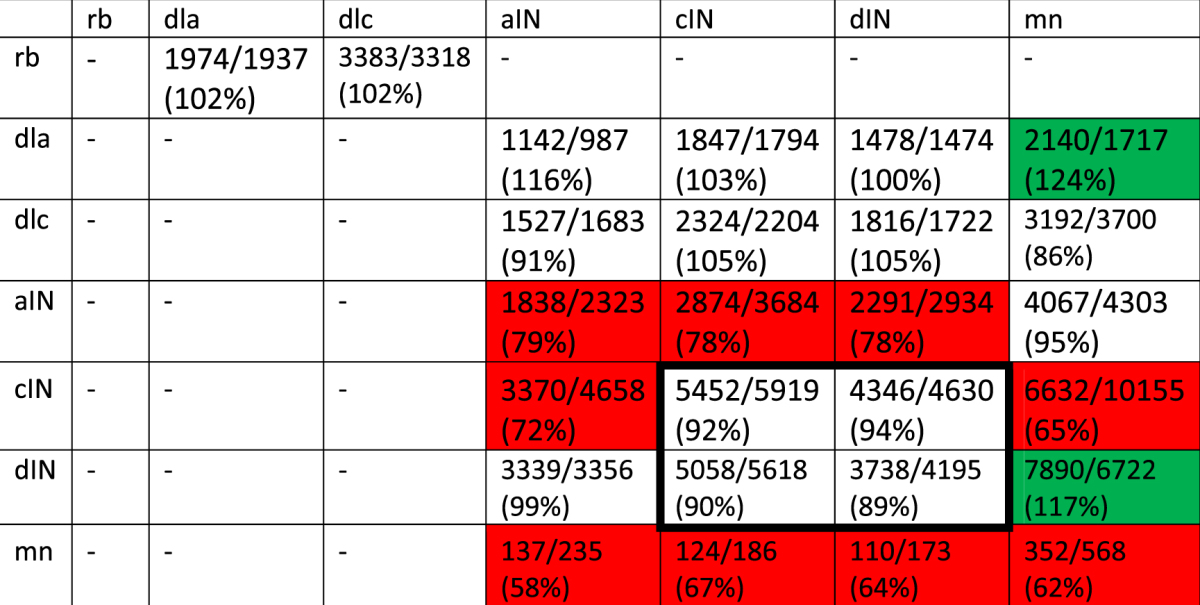
Number of synaptic connections (rounded average across 12 connectomes for each case) between different cell types on both sides, with and without fasciculation (first and second numbers respectively), as well as the percent change in synapse count that results from fasciculation.

Each row gives the number of connections made by neurons of a particular type (first column) onto neurons of every other type. Dashes indicate pairs where there is a negligible number of connections (<50) in both cases. Green and red highlights show pairs with particularly large increases and decreases (respectively) in synapse counts. The core CPG network (dINs and cINs) is highlighted with a bold border.

We can use Table 2 to study how each part of the network is affected by fasciculation. Synapse counts in the sensory pathway (RB→dli/dlc→CPG) are relatively unchanged, although it is interesting to note that fasciculation causes an increase in the strength of the ipsilateral reflex pathway from dlas directly onto motoneurons. Within the central pattern generator network, the biggest decreases in synapse counts are seen in connections to and from ascending interneurons (aINs), but since these neurons are almost completely silent during swimming this change cannot have an effect on the swimming pattern. Within the core “active” CPG network (cINs and dINs; bold bordered area in Table 2) the reduction in synapse counts is consistent but modest. However, we know that the characteristics of the swimming pattern generated by the core CPG network is very dependent on relative synaptic strengths; in the next section we will investigate the effect of fasciculation on the network’s spiking activity. Finally, fasciculation causes a marked increase in the strength of rhythmic excitation to motoneurons, thanks to an increase in dIN→mn excitation and a decrease in cIN→mn inhibition.

#### Fasciculation compensates for imperfect growth barriers

The axons of sensory RB neurons are confined to a narrow dorsal tract by two longitudinal barriers: one more ventrally at $y=\pm 127\mu m$ formed by the somata of dlc and dla neurons, and one more dorsally formed by the somata of RB neurons at $y=\pm 137\,{\rm{\mu }}m$. The majority of dlc and dla dendrites are located in this dorsal tract, so the barriers ensure that RB axons make many synapses onto these dendrites, resulting in a strong sensory pathway. In our model these barriers are implemented as hard barriers that axons can never cross, but in reality gaps in the barriers may be possible, for example due to spaces between somata. Can the “bundling” effect of fasciculation help to constrain RB axons to the dorsal tract even in the presence of holes in the growth barriers?

**Figure 8:**
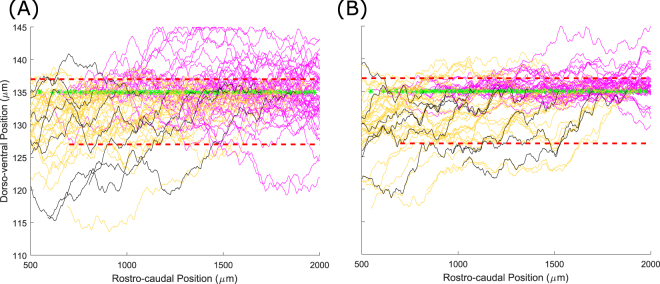
Fasciculation helps to constrain sensory axons to the dorsal tract when normal growth barriers do not perfectly block axon growth. Barriers (red lines) have 25 µm gaps at 25 µm intervals. Yellow lines show primary axons, magenta lines show secondary, black lines are pioneer primary axons. (**A**) With no fasciculation 24% of axon points are outside the marginal zone. (**B**) With fasciculation $(s=0.2,r=1)$, 15% of axon points are outside the marginal zone.

To answer this question we modified the growth model so that both barriers to RB growth contained 25 µm gaps at 25 µm intervals. We then grew RB axons as normal, and calculated what proportion of axon points lay outside of the dorsal tract. Figure 8A shows the results of this process in one case without fasciculation, demonstrating that RB axons are able to escape the dorsal tract via the gaps in the barrier. Figure 8B shows a simulation with fasciculation enabled $(s=0.2,r=1)$, showing that in this case axons are generally restricted to the dorsal tract despite the gaps in the barrier. Without fasciculation, 24% of RB axon points (std. 2.6, N = 12 simulations, 63 RBs in each) were outside the dorsal tract, whereas with fasciculation only 15% of points (std. 3.3, N = 12 simulations, 63 RBs in each) were (p < 0.001; Student’s unpaired t-test, two-tailed). The reason for this reduction can be seen intuitively, by considering that RB axon growth is primarily longitudinal. Since attraction causes growing axons to turn in the same direction as existing axons, pioneers and other already-grown axons all act as a form of soft barrier that prevents growing axons from growing in dorsal or ventral directions.

In most cases all of the pioneer axons remained in the dorsal tract, since the probability of any given axon escaping is relatively low. This laid down a scaffold that the follower axons followed. Fasciculation can therefore reduce the spread of axonal trajectories and help to restrict axon growth to a particular path or region.

#### Model optimization with fasciculation

**Figure 9:**
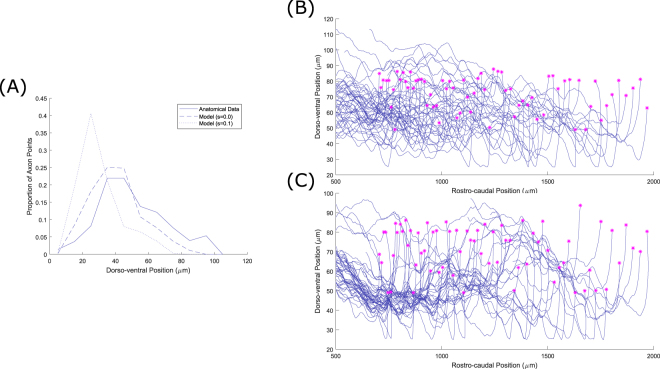
The effect of fasciculation on aIN axon trajectories. (**A**) Histogram comparing the dorso-ventral distribution of axons in the anatomical data used for optimization (solid line), optimized model output without fasciculation (dashed line) and model output with fasciculation sensitivity 0.1 (dotted line). (**B**,**C**) Example generated primary aIN axons for the s = 0 and s = 0.1 cases respectively. Magenta stars show positions where the axons begin growing.

As previously discussed, we performed the optimization process used to determine the axons’ sensitivities to the chemical growth environment without the presence of fasciculation. For a range of non-zero sensitivity values (from 0.1 to 0.7, $r=1\,\mu m$) the resulting pattern of axonal growth did not match the experimental data as well as when fasciculation was not present $(s=0)$. Figure 9 shows the effect of fasciculation on the dorso-ventral distribution of axon trajectories, which is the main component of the optimization procedure’s cost function. The data shown in this figure is for aINs, but we observed similar effects for other neuron types. From this it is clear that fasciculation has two main effects: a slight ventral shift in the modal axon position and a pronounced sharpening of the peak of the distribution. The ventral shift can be explained by the fact that axons initially grow ventrally before, at some point, turning to grow longitudinally. With fasciculation present, axons that might otherwise turn longitudinally at more dorsal positions are prevented from doing so by the influence of other axons. We explain the narrower peak in the distribution by noting that the axon data used for optimization was based on traced single axons from different individuals, and hypothesising that the wider peak in the anatomical data results from natural variation in the growth field / growth cue sensitivities between animals. If data from multiple axons in the same animal were available, these may show the effects of fasciculation as a sharper peak in the distribution.

We also repeated the optimization process using the same set of fixed sensitivity values in order to obtain new optimal parameters for the chemical gradients. Unsurprisingly this did not produce axons that more closely matched the anatomical data, as there was still a relatively tall and narrow peak in the distribution of dorso-ventral positions. This peak is a consequence of fasciculation causing axons to bundle together, which is not something that the optimization procedure can affect by changing growth cue sensitivities. Since the best fit to the limited anatomical data available is that produced when there was no fasciculation present during optimization, we use those optimized parameters throughout this paper.

### Effect of Fasciculation on Swimming Activity

As previously reported^4^, without fasciculation the growth model produces connectomes that very reliably generate swimming activity in response to sensory stimulation. In this section, we use a functional spiking model to investigate how fasciculation affects the activity generated by the network.

#### Effects of Fasciculation

**Figure 10:**
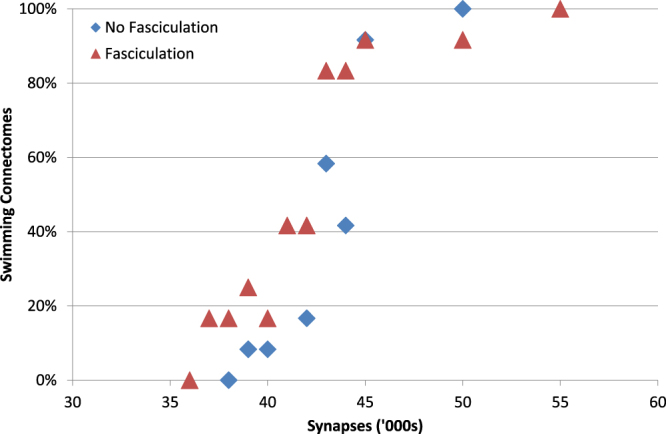
Reducing the number of synapses reduces the proportion of connectomes that can generate stable alternating patterns of MN spikes (swimming). A set of 24 connectomes (12 with fasciculation, 12 without) were modified to simulate the effect of reducing the synapse formation probability. Decreasing the probability (and therefore number of synapses) decreases the proportion of the 12 connectomes that can swim $(s=0.2,r=1)$. Each data point shows the average of N = 12 connectome simulations.

Introducing fasciculation reduces the number of synapses in a connectome. If fasciculation is too strong, the number of synapses is reduced so much that the generated connectomes are unable to swim. However, connectomes generated with only modest fasciculation (sensitivity 0.2, radius 1) do reliably swim in response to sensory input (6/6 connectomes), with a similar frequency (18.6 ± 0.8 Hz) to unfasciculated connectomes. This result raised the possibility that fasciculation would allow connectomes with fewer synapses than normal to still generate swimming activity. To investigate this we artificially lowered the probability of synapse formation in order to generate connectomes with reduced numbers of synapses. Swimming, defined as a stable pattern of alternating left and right motoneuron spikes at about 18 Hz, could be seen in the majority of connectomes even when the average synapse count was reduced to around 43,000. This threshold for swimming was roughly the same regardless of whether connectomes were generated with fasciculation or not, although fasciculated connectomes were able to swim with slightly fewer synapses (Fig. 10).

**Figure 11:**
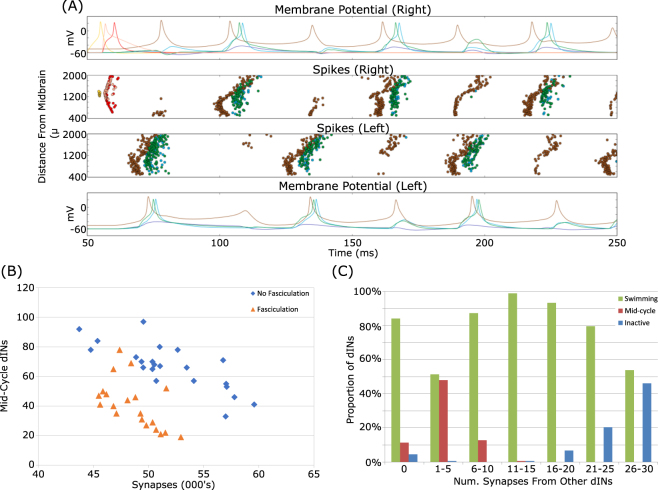
Fasciculation reduces mid-cycle dIN firing. (**A**) Simulation of a connectome with approximately 45,000 synapses and no fasciculation. A large number of dINs fire mid-cycle spikes at approximately the same time as neurons on the contralateral side of the body are active. Colour code as given in Table 1. (**B**) Reducing the number of synapses increases the number of dINs that fire mid-cycle spikes. For a given number of synapses, connectomes generated with fasciculation $(s=0.2,r=1)$ have fewer mid-cycle dINs. (**C**) A dIN’s spiking pattern depends on how many synapses it receives from other dINs. Neurons that receive little excitation (but not none) have a roughly 50% chance of firing mid-cycle spikes. Inactive dINs tend to receive a lot of excitatory input, but are unable to spike due to depolarisation blockade. Data from four connectomes with fasciculation and probability reduced to give approximately 50,000 synapses.

However, in connectomes without fasciculation but with fewer synapses there was a much greater tendency for dINs to fire mid-cycle spikes, i.e. at roughly the same time as contralateral neurons (Fig. 11A). This happens because dINs are capable of firing NMDA-driven pacemaker spikes after their normal post-inhibitory swimming spikes. Normally these spikes do not occur, since contra-lateral inhibition arrives early enough to stop them, but decreasing excitatory synaptic drive to a dIN decreases the delay to the first NMDA-driven spike. If this delay is short enough (i.e. when the number of synapses have been reduced dramatically) then spikes can happen before commissural inhibition arrives. This aberrant activity is not seen in real swimming, and we therefore consider swimming in which there is a lot of mid-cycle dIN activity to be lower quality. By this measure, fasciculation allows the same quality of swimming to be achieved with fewer synapses (Fig. 11B).

Fasciculation’s effect of reducing the number of mid-cycle dINs is somewhat surprising, since fasciculation also reduces the number of synapses. This apparent contradiction is explained by the fact that dINs that do not receive any excitatory input from other dINs rarely fire mid-cycle spikes (Fig. 11C). Fasciculated connectomes tend to have many more dINs that do not receive any synapses from other dINs (non-fasciculated: 6 ± 3 dINs, fasciculated: 31 ± 6 dINs, 24 connectomes, p < 0.001, Welch’s *t*-test). The spatially localised axon bundles that result from fasciculation mean that a dIN tends to either receive a lot of excitatory input or none at all, avoiding the intermediate levels of excitation that lead to mid-cycle spiking.

## Discussion

In this paper we present a new computational model of neuronal development in the *Xenopus laevis* spinal cord that includes the effects of axon-axon interactions. Our results suggest that attraction between axons (fasciculation) produces networks that have a simpler structure than networks without fasciculation, and that can generate reliable swimming behaviour with fewer synapses. Fasciculation also helps to produce appropriate patterns of axon growth when other growth cues (here the marginal zone barriers) are damaged. As discussed in section “Model optimization with fasciculation”, the model was optimized to match a set of measured axons that were taken from different individuals, which potentially means that axon positions in this data are more widely spread out than they would be within a single animal. When fasciculation is included in the model the spread of axon positions becomes narrower, and we have shown that the more selective connectivity that results increases the reliability of swimming. This highlights a potential important limitation of combining measurements of single axons from different animals together into a single dataset.

We are aware of two other computational models of axon fasciculation in the literature. The model described in^15^ simulates axon growth as a random walk on a lattice. This model includes the ability for axon type dependent interactions (e.g. axons may be attracted to the same type but repulsed by a different type), and while this feature was not included in our model for reasons of computational efficiency it is something we plan to investigate in future work. Their paper also includes a process whereby axons can detach from a fascicle (i.e. when they have reached a target region); our model does not explicitly include a mechanism for this but does allow axons to detach from bundles naturally, for example if the concentration of growth cues becomes strong enough to overcome the attractive force. The advantage of the model in^15^ is that its reasonably simple mathematical formulation allows theoretical results (e.g. about fascicle size) to be calculated, whereas we have followed a more experimental approach. A relative strength of our model is that it is built upon on an existing model of axon growth in the tadpole spinal cord that already includes other biologically realistic features of the growth environment, such as growth cue gradients and barriers.

In^14^ a model is presented that is closer to ours in terms of its implementation, as it features axons that grow in continuous space in response to external growth cues (but without barriers). The model simulates the effects of cell adhesion molecules that allow touching axons to join together, and includes chemoattractants that diffuse from axons to attract other nearby axons. In this model they found that the strength of environmental growth cues was not generally strong enough to cause axons to unbundle from a fascicle, so they allowed axons to switch to repulsing each other once the external growth environment reaches a certain trigger threshold; a mechanism that is biologically motivated. We have done some preliminary work in this direction, whereby commissural neurons switch from attraction to repulsion once they have crossed to the other side of the body, but we will present these results more fully in a later paper. An advantage that our model has over both of the previous models described here is that it produces complete tadpole spinal connectomes that can be simulated using a spiking network. The results of these spiking simulations can be compared with known biological results, in order to study the functional effect of fasciculation.

As with the previous models, ours can be used to investigate physical properties of fasciculation, including sorting dynamics and the mechanics of fascicle formation. Despite the simplicity of the attraction/repulsion model, we are still able to visually observe the tendency for axons to grow together, even in the case of relatively weak attraction. With some further refinement it would be possible to use the model to study the effect of varying the growth cone size/axonal interaction distance on the formation of fascicles. This question is relevant to understanding the pathobiology of diseases of abnormal axon pathfinding and subsequent synaptogenesis, such as autism^12^, as protein mutations that alter growth cone size could alter fascicle formation patterns and subsequently impact on correct axon pathfinding. These results would also be relevant to the design of regenerative therapies that treat peripheral nerve injuries, predicting, for example, that growing neurons with large growth cones along pre-laid axon tracts would lead to the formation of fewer, larger fascicles. This would be desirable if the goal were to grow a fascicle of axons towards a single target area. To investigate this using our model, a modification could be made such that nearby axons initially grow towards each other, before growing in along parallel trajectories when very close.

The results presented in this paper show that there are at least two plausible roles for fasciculation: reducing the number of synapses required for reliable swimming and improving RB axon guidance when growth barriers are damaged. This latter result is at least partially in agreement with the finding that the formation of a longitudinal tract by RB axons is disrupted when expression of cell adhesion molecules related to fasciculation is disrupted^7^. Conversely, however, it has been suggested that fasciculation may make aberrant axonal growth worse in some situations, since in *Drosophila* embryos with disrupted growth barriers follower axons may grow far away from their target areas as a result of fasciculating onto pioneers^23^. It is difficult to assess whether the patterns of axon growth that our new model generates match biological spinal cord axons more closely than when fasciculation is not included. The data available to us about spinal axons for developmental stage modelling (stage 37/38) are generally from studies where only single neurons were stained, which makes it impossible to discern bundles. Evidence from tadpoles at an earlier stage of development does suggest that fasciculation is a feature of axon growth for both RB neurons^18^ and commissural neurons^21^; however it is possible that as the animal grows fasciculated axons are pulled apart, which would again lead to bundles not being present at later stages. Our model does not include any simulation of axon shaft dynamics (i.e. how axons move after their initial growth), meaning it is unable to show the effects of further body growth on axon position, or reproduce the “zippering” phenomenon studied in^16^. Furthermore, there are hypothesised advantages to fasciculation which our model has not explored, such as it leads to more efficient and targeted network formation^9^. Nevertheless, our model suggests that fasciculation could play other important roles in axon guidance in the tadpole spinal cord. It should also be noted that our approach is general and can be applied to simulating the development of other nervous systems including humans. A greater understanding of how nervous systems develop and recover from damage will hopefully one day lead to new treatments for developmental disorders and traumatic injuries.

### Data Availability

The code used to perform the experiments described in this paper is available from the authors on request.
